# Supplementary material for: The potential impact of vaccine passports on inclination to accept COVID-19 vaccinations in the United Kingdom: Evidence from a large cross-sectional survey and modeling study
Source: eClinicalMedicine. 2021 Sep 9;40:101109. doi: 10.1016/j.eclinm.2021.101109 (PMC8428473; doi:10.1016/j.eclinm.2021.101109)
Supplement: Supplementary file 1 [file mmc1.docx]

There is only one supplementary file. This is our supplementary appendix (appendix_9-aug-21.doc).

The caption for this file can be:

Supplementary appendix: The supplementary appendix contains supplementary methods and figures that accompany the maintext.
